# Reply to Leong et al. Comment on “Hosseini et al. Effects of Vitamin D Supplementation on COVID-19 Related Outcomes: A Systematic Review and Meta-Analysis. *Nutrients* 2022, *14*, 2134”

**DOI:** 10.3390/nu15010060

**Published:** 2022-12-23

**Authors:** Banafshe Hosseini, Asmae El Abd, Francine M. Ducharme

**Affiliations:** 1 Clinical Research and Knowledge Transfer Unit on Childhood Asthma, Research Centre, Sainte-Justine University Health Centre, Montreal, QC H3T 1C5, Canada; 2 Department of Pediatrics, Faculty of Medicine, University of Montreal, Sainte-Justine Hospital, Montreal, QC H3C 3J7, Canada; 3 Department of Social and Preventive Medicine, Public Health School, University of Montreal, Montreal, QC H3N 1X9, Canada

We thank Drs. Leong et al. for their comments [1] and feedback on our article [2]. In their commentary, Leong et al. make the criticism that our findings were based on data from both randomized controlled trials (RCTs) and non-randomized studies of intervention (NRIS), and our findings are in disagreement with a previous meta-analysis conducted by Stroehlein et al. [3] Leong et al. also states that our methodological quality assessment was not clear for one of the included RCTs [4], previously conducted in our group. In this response, we show that while we included both RCTs and NRIS, our meta-analysis was stratified by the study design, and protective effects of vitamin D supplementation on the intensive care unit (ICU) admission rate as well as the need for mechanical ventilation was observed in both RCTs and NRIS as well as pooling all designs together. Furthermore, here we also provide additional clarifications regarding our methods and criteria for assessing the quality of the included studies.

As mentioned in the original paper, while we included data from both RCTs and (NRIS in our meta-analysis, all studies were stratified by the study design. As presented in Figures 2–4 in the original paper, the test for subgroup differences the based on study design was not significant, which implies the fact that the magnitude of benefit was not significantly different in the RCTs and NRIS. Also, throughout the paper, results were described based on the study design, and if the effect did not reach statistical significance in any of the subgroups (RCTs or NRIS), this was clearly stated in the relevant section. We also acknowledged this limitation along with others in the discussion section. We also acknowledged that studies varied in terms of study design, participants (baseline 25(OH)D, severity of COVID-19), and intervention (dose, regimens, duration). However, we addressed this by conducting several a priori specified subgroup analyses to shed some light on the impact of baseline serum vitamin D status and dosing regimens on the magnitude of effect. 

Methodological quality of RCTs was assessed by the Cochrane Handbook risk of bias tool. Regarding our RCT on vitamin D and risk of COVID-19 in healthcare workers [4]: random sequence generation was used; it was a triple-blinded randomized controlled trial, with no evidence of unblinding throughout the intervention; data for any outcome was available for all of randomized participants; methods of measuring outcome were described thoroughly; and we did analyze our results based on a pre-specified analysis plan that was finalized before unblinded outcome data were available for analysis. Therefore, the overall risk of bias was determined as low. Furthermore, the study quality assessment was conducted independently by two authors (BH and AEA).

Our meta-analysis showed that vitamin D supplementation, administered in hospitalized COVID-19 patients, is associated with a significant reduction in ICU admission, and need for mechanical ventilation, as observed in both RCTs and NRIS, and when pooling all designs. We also showed that vitamin D supplementation was significantly associated with a reduced risk of mortality, as confirmed in NRIS, and when pooling all designs. Our findings are in line with a previous meta-analysis of 13 studies, which concluded that vitamin D supplementation was significantly associated with a lower risk of ICU admission and mortality in COVID-19 patients, with an effect size comparable to what we found in the present study [5]. However, our results do not appear to be consistent with findings from a a living systematic review conducted by Stroehlein et al. [3], which was last updated in May 2021. Several additional intervention studies have been published since June 2021, which shed more light on the role of vitamin D supplementation in COVID-19.

Nevertheless, as we concluded in our paper, an updated meta-analysis upon completion of ongoing trials is needed to expand our understanding of the effects of vitamin D supplementation on preventing and managing COVID-19.
